# Accidents Associated with Bathing in Home Care Services for the Aged in Japan

**DOI:** 10.2188/jea.11.139

**Published:** 2007-11-30

**Authors:** Shinya Hayasaka, Masanobu Okayama, Shizukiyo Ishikawa, Yosikazu Nakamura, Eiji Kajii

**Affiliations:** 1 Department of Community and Family Medicine, Jichi Medical School, Tochigi, Japan.; 2 Department of Public Health, Jichi Medical School, Tochigi, Japan.

**Keywords:** baths, accidents, home care services, aged, Japan

## Abstract

Objectives: To reveal what kinds of accidents happen associated with bathing in home care services for the aged in Japan.

Methods: The study was cross-sectional in design. In November 1999, a postal questionnaire survey was conducted of 828 councils selected from the list of the National Council of Social Welfare of Japan by a systematic sampling method (extraction rate was 25%). The main outcome measures were characteristics of cases of accidents associated with bathing service for the aged, including patient age, sex, time of occurrence, symptoms, and results of accidents.

Results: Replies were received from 683 (82%) councils. Of the councils that replied, 430 (63%) reported providing bathing service for the aged. Of these 430 councils, 108 (25%) have experienced accidents, and 130 cases were analyzed. Affected patients had a mean age of 80.7 years (SD: 9.0 years), and 71 were females (55%). Sixty-two (48%) had symptoms of a disease or had accidents after bathing, and 42 (32%) presented with loss of consciousness. In results of accidents, 14 (11%) were reported to have died.

Conclusion: This study revealed that there were not a few accidents associated with bathing in home care services for the aged in Japan, the most frequent symptom was loss of consciousness, most accidents occurred after bathing, and that some patients died as a result of bathing provided by home care services.

## INTRODUCTION

In Japan, the number of aged individuals requiring help to live is increasing^1^^)^. At present, about 700,000 people aged 65 years or older need help with bathing because of functional deficits^2^^)^. The Japanese national government presently provides bathing services supported by public care insurance. This insurance system has been applied to the aged by the government since April 2000. Bathing is a major service of home care services in Japan.

The Japanese have been fond of soaking in a hot tub up to the neck for hygiene and relaxation since the 17^th^ century. Today, most Japanese people take baths in this style daily. Therefore, the aged also obtain satisfaction only with this bathing style, and are not satisfied with a shower. However, in this bathing style, warm water temperature and water pressure make an overload on the whole body^3^^)^. Therefore, many accidental deaths of the aged due to bathing have been reported. In Tochigi Prefecture, 1,348 persons drowned in bathtubs from 1978 to 1992, and the mortality rate per 100,000 persons was 251 for the 70-79 age group, and 469 for those 80 and over^4^^)^. On the other hand, there have been few reports of accidents due to bathing provided by home care services for the aged.

To reveal what kinds of accidents occur in association with bathing in home care services, we conducted a survey by mail throughout Japan.

## MATERIALS AND METHODS

### Study design

This study was cross-sectional in design.

### Procedure and sampling

In November 1999, a postal questionnaire survey was conducted with 828 councils selected from the list of the National Council of Social Welfare of Japan^5^^)^ by a systematic sampling method. Every fourth council was sampled, yielding an extraction rate of 25%. A council of social welfare is a public organization that provides various home care services to the aged. All cities, towns and villages of Japan have by law established such councils. We requested cooperation with the survey by phone if no reply had been received by three weeks after mailing.

### Questionnaire

Before preparing the questionnaire, we met directly or phoned the staff of six optional councils to perform interviews in July 1999. We asked the staff what kinds of accidents occurred in association with bathing services. Based on their opinions, we prepared a questionnaire including the following items: 1) Do you provide bathing services? ; 2) Have you experienced (an) accident(s) (including injury and sudden-onset illness) that the staff considered related to bathing service until now? If answers to 1) and 2) were “yes”, we asked the following as well: 3) What kind of symptom or situation resulted from the accident you experienced? ; 4) When did the accident happen? ; 5) What result of the accident happened in the patient? We did not determine a period to the accident about occurrence.

In September 1999, for the purpose of improving the quality of this questionnaire, we carried out a pilot study of 37 nursing homes in Tochigi Prefecture, and examined the contents of the questionnaire again. We then conducted a mailing survey to the sampled councils throughout Japan using the improved questionnaire.

### Analysis

Patients with accidents were classified by symptom according to the following method. One investigator (S.H.) made a list of the category of symptoms from the answers. Then, two other investigators (M.O. and S.I.) independently classified these symptoms using the list. In cases of disagreement on classification, the consensus of the investigators was obtained after discussion.

Patients were categorized three groups by time of accident occurrence. The categories by time of accident occurrence were; before, during, and after bathing. We observed the numbers of patients, and used chi-square tests for categorical comparisons of sex and results of accidents. Difference in the means of age was tested by one-way analyses of variance.

Statistical analyses were performed with the statistical program SPSS 10.0J for Windows (SPSS Inc., Chicago, U.S.A.). A p value of less than 0.05 was considered to indicate statistical significance.

## RESULTS

Of the 828 surveys mailed, 683 (response rate 82%) were returned. Nine councils replied by telephone, and we included these councils in the analysis. Of the 683 councils, 430 (63%) reported providing bathing services for the aged.

Of these 430 councils, 108 (25%) had experienced accidents, and 134 cases were reported. We excluded four cases from analysis because they could not be identified as accidents on discussion for classification of symptoms. We thus analyzed 130 cases (Table 1).

**Table 1.** Characteristics of cases of accidents associated with bathing service for the aged categorized by time of occurrence.

Characteristics	Total		Before bathing		During bathing		After bathing		Not reported	
	n (%)		n (%)		n (%)		n (%)		n (%)	
n	130	(100)	18	(14)	46	(35)	62	(48)	4	(3)
Age (Year, Mean±SD)	80.7	±9.0	81.7	±7.6	79.8	±9.5	81.1	±9.3	81.3	±5.5
-49	1	(1)	0	(0)	1	(100)	0	(0)	0	(0)
50-59	3	(2)	0	(0)	0	(0)	3	(100)	0	(0)
60-69	9	(7)	1	(11)	4	(44)	4	(44)	0	(0)
70-79	35	(27)	7	(20)	13	(37)	14	(40)	1	(3)
80-89	55	(42)	6	(11)	21	(40)	26	(49)	2	(4)
90-	23	(18)	4	(17)	6	(26)	13	(57)	0	(0)
Not reported	4	(3)	0	(0)	1	(25)	2	(50)	1	(25)
Sex										
Male	56	(43)	8	(14)	22	(39)	25	(45)	1	(2)
Female	71	(55)	10	(14)	23	(32)	36	(51)	2	(3)
Not reported	3	(2)	0	(0)	1	(33)	1	(33)	1	(33)
Circumstances of accidents										
Symptom										
Loss of consciousness	42	(32)	4	(10)	12	(29)	26	(62)	0	(0)
Pallor of the face and/or cyanosis	29	(22)	4	(14)	9	(31)	15	(52)	1	(3)
Hypotension	24	(18)	6	(25)	2	(8)	16	(67)	0	(0)
Nausea and/or vomiting	12	(9)	0	(0)	4	(33)	6	(50)	2	(17)
Dyspnea	11	(8)	1	(9)	6	(55)	3	(27)	1	(9)
Hyposthenia	9	(7)	0	(0)	4	(44)	5	(56)	0	(0)
Convulsion	8	(6)	1	(13)	4	(50)	3	(38)	0	(0)
Rush of blood to head (“Nobose” in Japanese)	7	(5)	0	(0)	3	(43)	4	(57)	0	(0)
Arrhythmia and/or tachycardia	5	(4)	0	(0)	2	(40)	3	(60)	0	(0)
Elevation of blood pressure	4	(3)	0	(0)	1	(25)	3	(75)	0	(0)
Discomfort	4	(3)	0	(0)	1	(25)	3	(75)	0	(0)
Incontinence of urine and/or feces	4	(3)	0	(0)	4	(100)	0	(0)	0	(0)
Asphyxia	4	(3)	0	(0)	1	(25)	3	(75)	0	(0)
Cardiac arrest	4	(3)	0	(0)	1	(25)	3	(75)	0	(0)
Others	17	(13)	1	(6)	5	(29)	11	(65)	0	(0)
Disease										
Cerebral ischemic disease	10	(8)	1	(10)	5	(50)	4	(40)	0	(0)
Ischemic heart disease	2	(2)	0	(0)	0	(0)	2	(100)	0	(0)
Cerebral hemorrhage	2	(2)	0	(0)	0	(0)	2	(100)	0	(0)
Others	3	(2)	0	(0)	2	(67)	1	(33)	0	(0)
External causes										
Injury	13	(10)	5	(39)	7	(54)	1	(8)	0	(0)
Falling	10	(8)	2	(20)	5	(50)	3	(30)	0	(0)
Iatrogenic trouble	4	(3)	1	(25)	2	(50)	0	(0)	1	(25)
Bleeding from decubitus	1	(1)	1	(100)	0	(0)	0	(0)	0	(0)
Unclear	2	(2)	0	(0)	0	(0)	2	(100)	0	(0)
Results of accidents										
Observation by council staff										
Recovery	38	(29)	4	(11)	15	(40)	19	(50)	0	(0)
Death before consulting a physician	4	(3)	0	(0)	4	(100)	0	(0)	0	(0)
Consult a physician										
Recovery	52	(40)	7	(14)	17	(33)	27	(52)	1	(2)
Death	10	(8)	2	(20)	2	(20)	5	(50)	1	(10)
Result was not reported after consulting a physician	24	(18)	5	(21)	7	(29)	11	(46)	1	(4)
Not reported	2	(2)	0	(0)	1	(50)	0	(0)	1	(50)

Cirumstances of accidents: check that all apply.

Sixty-two (48%) patients presented symptoms of diseases or had accidents after bathing, and 46 (35%) accidents happened during bathing. Of the 62 patients, 28 patients had accidents in mean 37.4 minutes (standard deviation: 40.4 minutes, range: three to 180 minutes) after bathing. Exact times of occurrence were not reported in other 34 patients. The patients had a mean age of 80.7 years (standard deviation: 9.0 years, range: 45 to 97 years). The difference of mean age among groups categorized by time of occurrence was not statistically significant (p = 0.87). There were 71 females and 56 males. The difference between the sexes were not significant (p = 0.11).

By symptom, 42 (32%) patients had loss of consciousness, 29 (22%) pallor of the face or cyanosis, and 24 (18%) hypotension. Concerning diseases and external causes, 10 (8%) patients suffered cerebral ischemic disease and 13 (10%) suffered an injury. On the other hand, in results of accidents, 52 (40%) consulted a physician and recovered, and 38 (29%) recovered with only observation by council staff. However, 14 (11%) patients died. Six of the 14 who died did so during bathing, and five did so after bathing. Of the six patients, four died before consulting a physician (p = 0.02).

## DISCUSSION

The present study revealed that 1) the most frequent symptom of bathing accident was loss of consciousness, 2) most accidents occurred after bathing, and 3) some patients with accidents died. Many deaths by bathing in the aged result from cerebral apoplexy^4^^)^, and bathing affects blood pressure, elevates blood viscosity, and may cause cerebral ischemic disease^6^^)^. For these reasons, we believe that many of 42 (32%) cases which presented loss of consciousness resulted from cerebral ischemia, and, notably, 10 (8%) patients were reported to have cerebral ischemic disease. These results suggest that home care staff should carefully monitor for loss of consciousness in bathing services.

Sixty-two (48%) accidents happened after bathing. This result suggests that it is important to observe the aged not only during bathing but also after bathing. It is said that bathing decreases blood pressure and increases blood viscosity for more than 12 hours^3^^, ^^6^^)^. In this study, it is not clear whether bathing caused all accidents. However, it is important that the long-term effects of bathing be recognized.

Further, 14 (11%) deaths were reported. Bathing services should thus be provided more safely. There are presently not guidelines for judgment of advisability of bathing for in the aged for use by home care services in Japan. Therefore, home care staff must judge whether bathing is possible, based on their own experience. We previously reported that 86% of councils need guidelines for judgment of bathing advisability^7^^)^. It will be necessary to determine the clinical details of these cases of death to ensure the safety of bathing services.

This study has certain limitations. There were potentially more accidents which actually happened than reported accidents. Because there was a bias that councils were disinclined to report accidents, and they did not reply to this questionnaire. We did not survey details of councils’ characteristics, making it impossible for us to analyze relationships between accident and council characteristics.

We sampled councils from the list of the National Council of Social Welfare of Japan by a systematic sampling method. Because this list has no recognizable pattern, the systematic sampling method is same as a random sampling method. In addition, this study’s response rate was 82% including councils providing no bathing service. For these reasons, we believe that our study has little bias in survey results and it yields information sufficient to provide an outline of bathing accidents.

In conclusion, this study revealed that there were not a few accidents associated with bathing in home care services for the aged in Japan, the most frequent symptom was loss of consciousness, most accidents occurred after bathing, and that some patients died as a result of bathing provided by home care services. Home care staff should carefully observe the aged not only during bathing but also after bathing.
